# Disorder at the Synapse: How the Active Inference Framework Unifies Competing Perspectives on Depression

**DOI:** 10.3390/e27090970

**Published:** 2025-09-18

**Authors:** Christopher G. Davey, Paul B. Badcock

**Affiliations:** 1 Department of Psychiatry, University of Melbourne, Melbourne, VIC 3010, Australia; 2 Centre for Youth Mental Health, University of Melbourne, Melbourne, VIC 3010, Australia; pbadcock@unimelb.edu.au; 3 Orygen, Melbourne, VIC 3052, Australia

**Keywords:** depression, active inference, psychotherapy, antidepressants

## Abstract

Depression is one of the most disabling of all disorders across the community, yet many aspects of the disorder remain contentious. Psychosocial and biological perspectives are often placed in opposition to one another, which in part reflects a failure of our explanatory frameworks. The active inference account of brain function breaks down this dualism, demonstrating that bodily processes are deeply integrated with the social world. It shows us that there is no contradiction in understanding depression as a product of the social environment at the same time as having a brain basis and manifesting in biological symptoms. From an active inference perspective, depression can be thought of as a synaptopathy: a disorder that arises from alterations to the excitatory-inhibitory balance enacted at the synapse, reflecting the interoceptive precision-weightings that have changed in the context of psychosocial instability. Therapies that alleviate depressive symptoms act at different levels of the active inference framework to re-weight precision estimates and the confidence we have in our predictions: this is true for psychotherapies, lifestyle interventions and antidepressant medications. Their effectiveness is often only partial, and while different treatment modalities can complement one another, there is a need for continued development of new and better treatment options.

## 1. Introduction

Depression is one of the most disabling of all disorders in the community. It is estimated to affect about 8% of adults each year, at a relatively consistent rate across cultures [1,2]. The first episode of depression is usually experienced in adolescence or early adulthood, and while the course is variable, it tends to recur throughout adult life [3]. Depression has significant effects on functioning at the individual level, and this, along with the long period of the life course over which it recurs, explains its substantial contribution to disability at a population level [4].

While the community-level impact of depression has gained increasing recognition, many aspects of the disorder remain contentious. Episodes of depression often appear to be responses to stresses and losses, and there is resistance to considering that it could therefore be related to underlying brain processes [5]. The major contributors to depression are often argued to be social factors, especially those that impose themselves because of a person’s poverty and marginalisation [6,7]. As a corollary, many commentators favour treatments that address social and psychological factors. The public health response to depression, it is argued, should be to address food and housing insecurity, unemployment, childhood maltreatment and other social determinants, thereby reducing the stresses that precipitate depression [8].

At the individual level, psychotherapy is an effective treatment for depression [9]. Lifestyle changes such as attending to diet and exercise can also be effective [10]. Research findings showing their effectiveness are often promoted in newspaper articles and other media, reinforcing the notion that depression arises as a result of unhealthy living, and can be addressed by helping people to become physically healthier [11,12].

Antidepressant medications are the most frequently used treatments for depression, used by about 1 in 8 adults in developed nations [13,14]. In contrast to psychosocial approaches to treatment, they are often reported in the media unfavourably [15,16]. Although there is good evidence that antidepressants are effective for depression [17,18], it is true that treatment effect sizes are relatively small, with a substantial minority of patients not obtaining significant benefit [19]. (Their effectiveness is similar, it should be noted, to other treatments for depression, and to treatments in psychiatry and medicine more generally [20]). The sense of suspicion about the promotion of medications as a treatment for depression reflects a deeper concern. Why would a person use a medication to cure an ill caused by our society? Why should we medicate what appears to be an understandable response to the vicissitudes of life?

Many perspectives on depression are held in opposition: biological versus social, brain versus mind, medication versus psychotherapy. They assume a dualistic understanding of mental life: that the brain and mind exist separately and reflect different processes [21]. In part, this dualist perspective reflects a failure of our explanatory frameworks. We have not had good theories about why biological symptoms like insomnia, reduced appetite and fatigue can arise from social precipitants.

There is a need then for a framework that can break down this dualism and show us that brain (and bodily) processes are deeply integrated with the social world. The active inference account of brain function proposed by Friston and colleagues [22,23] provides us with such a framework. By showing how the brain creates predictions about bodily processes in the context of the social environment, we can understand how extensively the two domains are integrated [24]. There is no contradiction in understanding depression as being a product of the social environment at the same time as having a brain basis and manifesting in biological symptoms. Our aim in this manuscript is to demonstrate how the active inference framework helps us to understand depression. We believe it can usefully address areas of contention among the community of depression researchers. We are keen to demonstrate how the framework allows us to break down the dualism that pervades the space: we can understand depression as both a disorder of the brain and a psychosocial disorder. Treatments can operate at different levels, but can be understood as being effective within the active inference framework.

## 2. Depression as a Response to Environmental Stressors

Depressive episodes are often precipitated by social stresses and losses, especially early in the course of illness [25]. These can include relationship breakups, job losses, interpersonal difficulties and financial stresses [26]. Such events will be more frequently experienced by people who live in communities with poverty, housing instability, high levels of use of alcohol and other drugs, and racial discrimination [8]. While these structural determinants are important, depression is experienced across the social strata [27]. Losses and disappointments are universal experiences: they are often navigated without significant effects on mental health, but for many people, depression arises in their wake.

Social stresses and losses create vulnerabilities across the lifespan. Early life stresses such as neglectful parenting and childhood trauma leave people vulnerable to depression as they enter adolescence [28]. The incidence of depression increases markedly after puberty, with adolescence representing a critical window of vulnerability [29]. The biological changes that accompany puberty are associated with increased sensitivity of young people to interpersonal contexts and significant changes in their social environments, with the development of more hierarchical peer group relationships and the beginning of intimate relationships [30,31]. In this context, there are significant increases in the number and intensity of socially salient stressful life events, which are major drivers of the increase in the prevalence of depression from adolescence [32]. Such stresses continue throughout adult life, with depressogenic factors such as loneliness becoming more important with age [33,34], with the result that a relatively high prevalence of depression persists across the lifespan [35].

The significant contribution that environmental factors make to depression is evident in twin studies. These show that environmental factors account for 50–70% of the variance for vulnerability to depression [36]; greater than their contribution to mental disorders such as schizophrenia, bipolar disorder and attention deficit hyperactivity disorder [37]. The environmental factors that are important for depression include not only those that operate in a person’s current environment (such as losses and stresses), but also those that have had their effects during development, including the childhood home environment, schooling and peer influences [38]. These early experiences increase susceptibility to negative life events and the onset of depression during adolescence and adulthood [39].

The role of proximal environmental factors is important. But for many people, episodes of depression are not precipitated by identifiable stressful life events. This is more likely to be the case for later recurrent episodes [25,40]. Episodes later in the course more often appear to arise autonomously, suggesting changes in the nature of the disorder over time [41].

## 3. Depression in the Brain and Body

It is clear that depression has some relationship with brain processes, with many studies showing changes in brain structure and function in people with depression compared to those without it [42,43,44]. The differences arise at the level of group comparison and are small overall, with the groups largely overlapping: brain imaging does not usefully discriminate those with depression from those without, and is not useful for diagnosis [45]. Brain imaging has shown, though, some consistency in the pattern of brain changes in depression, implicating subcortical regions such as the amygdala and hippocampus and midline frontal cortical regions [42,43,46,47].

Other biological differences are also evident. At a symptom level depression is associated with disturbances in sleep, appetite, psychomotor activity and energy levels. Curiously, symptoms can show changes in either direction: patients can experience insomnia or hypersomnia, increased or decreased appetite, and psychomotor slowing or agitation [48]. There can also be either decreased or increased energy levels, although we characterise the latter as being part of mania (or hypomania), which can accompany depression but in contemporary nosology forms a different illness—bipolar disorder [49].

There are group-level differences in inflammatory markers [50,51] and in measures of autonomic reactivity [52,53]. While genetic factors are less prominent for depression than for other mental illnesses, they do make a contribution. No individual genes confer substantial risk; many small-effect loci are hypothesised to operate through interacting biological networks to influence depressive vulnerability [54].

There is evidence that changes in the brain become more pronounced with depression recurrence. For example, hippocampal volumes become smaller [42], and there is more marked disturbance of the hypothalamic-pituitary-adrenal axis [55]. These disturbances, which persist after depression remission, set the groundwork for future episodes. Depression is argued to be a neuroprogressive illness, which provides an explanation for why later episodes of depression are more likely to arise autonomously rather than in response to stressful life events [55,56].

## 4. Antidepressants and Their Effects on Monoaminergic Systems

The first antidepressants were developed in the 1950s, and were demonstrated to exert their effects via their interactions with the brain’s monoaminergic systems [57,58]. Almost all of the antidepressants that have been developed since have worked on the same systems, albeit with greater specificity, and therefore fewer side effects—although without superior effectiveness [59].

The brain’s monoaminergic systems consist of three main networks, which use serotonin, norepinephrine and dopamine as neurotransmitters. These systems originate from regions in the brainstem and project widely throughout the cerebrum. They have neuromodulatory actions: they are inherently neither excitatory nor inhibitory, but rather modulate the excitatory and inhibitory actions of other neurotransmitters [60].

The most commonly prescribed antidepressants are the selective serotonin reuptake inhibitors (SSRIs), which include fluoxetine, citalopram and sertraline. These drugs bind to the membrane-bound serotonin transporter protein. The protein functions as a molecular channel to transport serotonin back into serotonergic neurons after its release [61]. SSRIs block this reuptake process, allowing more serotonin to accumulate in the synaptic space and to stimulate post-synaptic serotonin receptors, thereby influencing neural signalling patterns [62].

The observation that SSRIs exert their effects by increasing the amount of serotonin in the synaptic cleft led drug companies to claim that the medications were reversing a “chemical imbalance” [63]. This chemical imbalance theory of depression entered the public discourse, and has often been used as a shorthand justification for the use of antidepressants. There is little evidence, however, that there are differences in the levels of serotonin and its metabolites in people with depression [64]. There is clearly a more complicated story at play.

## 5. Non-Medication Treatments

The mainstay of depression treatments is psychotherapy. Cognitive behavioural therapy (CBT) is the most studied of these, but there are at least six other evidence-based psychotherapies for depression [65,66]. With CBT, the therapist works with patients to test and reconceptualise their aberrant beliefs about themselves, their social environments, and their futures; and encourages more social activity [67,68]. Other therapies, such as interpersonal therapy, focus more on improving the nature and quality of a person’s close relationships rather than their cognitive attributes; but similarly, the therapist encourages the patient to become more socially active rather than succumb to the tendency for social withdrawal [69]. The therapies are effective overall, but similarly to antidepressants, their effect sizes are modest [9].

Other approaches are effective in ameliorating depressive symptoms. Interventions that focus on addressing diet and exercise have been shown to reduce depressive symptoms [70,71]; although their effectiveness must contend with the difficulty people with depression have in engaging with prescribed health programs [72,73]. Anti-inflammatory medications can also be effective treatments—at least in the subset of patients who show evidence of inflammation [74,75].

## 6. The Active Inference Framework

The active inference framework of brain function, proposed by Friston, represents a fundamental reconceptualisation of how the brain works [22,23]. The theoretical framework builds upon a number of older ideas, including perceptual inference [76], cybernetics [77], predictive coding [78] and other Bayesian models of brain function [79,80]. Active inference is grounded in the principles of Bayesian probability theory, and describes the brain as an inference machine that actively generates and tests predictions about the world. Rather than passively processing sensory information, as in the classical cognitivist model, the brain instead generates models of the world that it continuously refines by minimising discrepancies between its prior beliefs about the body and its environment compared with incoming sensory data (i.e., it strives to minimise prediction errors). This occurs via two mechanisms: (i) by the brain updating its beliefs to better account for the sensory data, and (ii) by the brain instigating action to align its sensory inputs with its predictions [22,81].

**Figure 1:**
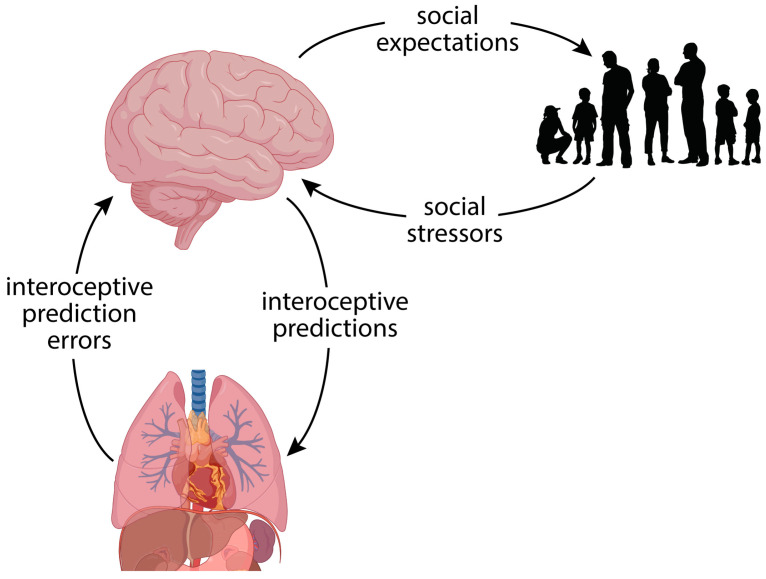
The active inference framework explains how the brain generates interoceptive predictions in the context of the social environment. Top-down predictions modulate autonomic and neurohormonal processes, while prediction errors arising from bodily states update the models. This bidirectional process is affected by environmental stressors that influence the precision-weighting of interoceptive prediction errors, providing an account of how psychosocial factors manifest in the biological symptoms of depression. Elements of the figure were created with https://www.biorender.com.

Of most relevance to depression, the active inference framework can be applied to interoceptive processes, whereby the brain generates predictions about expected bodily sensations and parameters. The nature of the active inference framework for interoception is provided in other manuscripts, which we recommend to readers who would like more detailed explanations [82,83,84,85]. We provide a summary here. In the active inference framework, bodily processes are understood as being managed according to environmental contingencies so as to optimise one’s capacity to survive and flourish. As in other domains, the brain generates predictions about bodily sensations, and where the sensations differ from the predictions, it either updates its prior beliefs, or changes visceromotor and neurohormonal output to align interoceptive sensations with its predictions [82,86]. Via these mechanisms, the brain provides constant adjustments to parameters associated with processes such as cardiorespiratory output, inflammation, sleep and appetite, adapting them to environmental demands (Figure 1).

The active inference framework is inherently hierarchical. Prior (Bayesian) beliefs generated at higher brain levels are applied to lower levels in a top-down manner. (Note that in active inference, a “belief” is a formal mathematical construct that refers to neuronally encoded probability distributions about the causes of sensory data, and should not be confused with “beliefs” in a folk psychology sense [87]). Prediction errors ascend in the opposite direction, modifying and refining predictions from the bottom up [22]. Higher level predictions are more general in nature, integrating predictions across multiple domains, such as the broad shape of interoceptive processes in the context of the social environment [24]. The predictions become more specific as they descend, generating expectations related to parameters such as heart rate, plasma osmolality and carbohydrate craving. In a similar vein, the prediction errors that arise from the bottom of the hierarchy are generated in specific sensory domains, informing lower-level predictions initially, and if they are not able to be explained away (e.g., by reflex arcs), they ascend the hierarchy to inform more general higher-level predictions [82,83].

## 7. Model Confidence and Precision Weighting

The influence of top-down predictions and bottom-up prediction errors exists in dynamic tension. The nature of this balance reflects model confidence. When confidence in prior beliefs is high, prediction errors are explained away at lower levels by top-down influences. In the interoceptive domain, this is hypothesised to occur via reflex arcs and neurohormonal allostatic loops, which are enacted without conscious awareness [83,88]. When confidence in higher-level priors is low, however, there is greater reliance on prediction errors to attune the brain’s models [81]. The prediction errors ascend the predictive hierarchy and lead to belief updating, often entering conscious awareness: interoceptive prediction errors become known to us as feelings (e.g., of hunger, anxiety or fatigue) [88].

The balance between top-down predictions and bottom-up prediction errors is enacted by precision-weighting. Neural signals are either upweighted or attenuated as a reflection of model confidence. This weighting is enacted at the synapse. Neurotransmitters such as serotonin, noradrenaline and dopamine act by modulating the influence of excitatory and inhibitory neuronal signalling, which are enacted by glutamatergic and GABAergic neurons, respectively [89,90]. While monoamine neurotransmitters provide rapid modulation, other systems are also implicated. The excitatory-inhibitory balance can be influenced by activation of glutamatergic NMDA receptors and by the action of neuropeptides [91,92]. The dynamic changes in precision-weighting enacted by these mechanisms are made more enduring by the process of long-term potentiation, whereby the strength of synaptic connections is altered via structural changes to the neurons [93].

## 8. An Active Inference Account of Depression

In viewing depression through the lens of the active inference framework, the social environment has particular significance for how interoceptive processes are managed [24,94]. By its nature, the social world is complex and often unpredictable. The stability of the environment—and our confidence in it—is reflected in the confidence we have in our internal models. When we have confidence in the future, the biological rhythms that underpin sleep, appetite and energy levels maintain their usual consistency.

When depressed, these interoceptive processes are altered. Depression is often precipitated by changes in a person’s social environment, with losses and stresses reflected in reduced confidence in one’s beliefs about the world [24,85]. Depression emerges when there is both uncertainty in one’s ability to successfully predict and respond to the social environment, along with high confidence in this uncertainty. A depressed mood instantiates hyperpriors that encode the expected long-term average of short-term fluctuations in precision by determining new set-points for neuromodulatory mechanisms that govern the sensitivity of our responses to prediction errors [95]. In other words, moods—as hyperpriors—determine the precision attributed to the confidence we have in our lower-order, more rapidly fluctuating emotional states [85]. With depression, a self-reinforcing negative affective state develops that is resistant to updating [96].

The symptoms of depression reflect changes in the way that interoceptive processes are subsequently managed [85]. There is a changed array of emotions, with more anxiety, irritability and tension, along with changes to longer-term physiological processes that manifest in disturbed sleep, altered appetite and fatigue [85]. There are shifts in behaviour, with withdrawal from social activities, which are anticipated to be aversive [24,94]. These changes occur via alterations in precision-weighting, which provides a mechanism for events in the social environment to influence biological processes. The active inference framework can therefore be seen to unify competing perspectives of depression, allowing it to be socially influenced as well as inherently biological.

## 9. Depression as Synaptopathy

From an active inference perspective, depression can be thought of as a synaptopathy. Synaptopathies were initially defined as neurological disorders that arose from genetic mutations of synaptic proteins, such as in Huntington’s disease [97]. It has more recently been applied to disorders where the primary deficit is assumed to be at the synapse, even though no precise causal deficit in synaptic protein function has been identified: these include mental disorders such as schizophrenia, autism and depression [98,99,100]. An active inference account of depression suggests that it arises from alterations to the excitatory-inhibitory balance enacted at the synapse. This balance is shifted by the action of neuromodulators and by neuroplastic changes, which reflect the interoceptive precision-weighting that has changed in the context of psychosocial instability, and manifest in the symptoms of depression [85]. It is the precision-weighting enacted at the synapse that is critical.

To be clear, the concept of the synapse in this account is shorthand for the hierarchical networks that consist of many billions of synapses; the coordinated, dynamic, extraordinarily complex activity of which underpins prior beliefs and the precision-weighting of prediction errors. In considering depression as a synaptopathy, we do not imply that depression can be reduced to brain function: while active inference processes might ultimately be manifested at the synapse, they incorporate higher-level beliefs about the complex social environment, and reflect the interpersonal and broader social milieu that provide the context for interoceptive processes [85]. Expectations about future experiences are influenced by past experiences, and are encoded at a synaptic level. That later recurrent episodes of depression are less obviously precipitated by proximal psychosocial stresses suggests that aberrant precision-weighting accrues from past experiences—both from developmental stresses and from previous episodes of depression. They leave evidence of their occurrence in their influence on precision-weighting models, creating a susceptibility to depression even in the absence of social precipitants.

## 10. Treatments Target the Synapse

The active inference conception of depression provides insights into how treatments for the disorder work. Depression arises from the complex re-weighting of interoceptive precision, and treatments for depression aim to modify this re-weighting [85]. This is more obvious for antidepressant treatments, but other treatments can also be seen to operate at the synapse: they re-weight precision estimates and improve model confidence [101].

Psychotherapies directly address higher-order models of the self and the confidence a person has in their interpersonal environments [102]. The active inference model of depression described here emphasises the role of future social expectations, with the success of therapies deriving from their ability to help patients scaffold a future that they can have more confidence in [85]. This can be related to the explicit goals of the therapy. In CBT, for example, correcting cognitive distortions about the future is one of its aims [67,68]. The therapist might encourage the patient to consider what changes they might make to their lives to instil a greater sense of stability and confidence; and can indirectly instil confidence by providing a safe and supportive environment in the therapy sessions themselves [103]. The behavioural component of CBT revises priors by encouraging the exposure of depressed patients to corrective environmental (particularly interpersonal) stimuli [104].

Interventions that aim to improve the lifestyle factors that contribute to depression—such as those that improve diet and exercise—can be seen to target the interoceptive prediction errors themselves as a means of alleviating depression by affecting depressogenic processes related to oxidative stress, inflammation and gut microbiota, among others [105,106]. Similarly, the treatment of depression with anti-inflammatory medications targets the aberrant inflammatory processes that represent, in the active inference framework, unresolved prediction errors [96].

Medications that affect the influence of monoamines have been the mainstay of antidepressant treatments and are targeted more directly at the synapse. As discussed, SSRIs act by modifying the amount of serotonin available in the synaptic cleft. While levels of synaptic serotonin are evident after the first dose, the antidepressant effects of SSRIs are not felt for a week or longer [107]. The mechanisms that underlie the effectiveness of SSRIs remain incompletely understood, but the delayed onset of effectiveness corresponds with changes in gene expression and the synthesis of new proteins [108,109]. These changes support neuroplastic processes such as the re-weighting of synaptic connectivity strengths and the formation of new synaptic connections [110]. The evidence for such SSRI-induced neuroplasticity is mainly drawn from animal studies, with only a few studies showing evidence of this in humans [111,112]. This limited evidence is supplemented by indirect evidence from human structural MRI and post-mortem studies that show morphological changes in regions such as the hippocampus [113].

Other antidepressant treatments have effects with quicker onsets. Low-dose ketamine, for example, acts directly on glutamatergic NMDA receptors and has an almost immediate antidepressant effect that is maintained for up to a week after a single dose [114]. It has neuroplastic effects, promoting synaptogenesis in prefrontal cortex and hippocampus in the hours to days after a dose [115].

Interventions target different aspects of depression, but their effects are experienced across levels. Interventions that address diet and exercise are targeted at lower-level somatic components at the same time as addressing the lifestyle factors that are embedded in the social environment. Psychotherapies target psychological and behavioural processes, but they have biological effects too: they have been demonstrated to change brain function [116]. And while medications operate at the synapse, the act of prescribing them has psychosocial aspects. They are provided in a therapeutic context, embedded in the supportive and predictable relationship between the patient and their prescribing clinician [117]. One of the reasons that SSRIs show only a small benefit compared to placebo treatments in clinical trials is that depression shows robust response to placebo pills, suggesting that much of the effectiveness of antidepressant medications can be ascribed to the therapeutic relationship and the act of prescribing [118].

Many patients do not respond to first-line treatments, whether that be to medications or psychotherapies. If medications work by adjusting precision-weighting in ways that often see improvement in depressive symptoms, they do so only approximately: for some patients more than for others (who might not notice any improvement, or even deterioration). Combining medication with psychotherapy and lifestyle modification might see aberrant precision-weighting nudged incrementally towards their previous long-term averages, with each operating at different levels of the predictive framework and having effects that are complementary.

## 11. Conclusions

Depression is a complex and disabling disorder that affects many people in the community. The diagnosis and its treatment remain contentious, in large part because we maintain a dualistic understanding of it. Many people view depression as an understandable response to environmental stresses, and are antagonistic towards accounts that privilege brain processes and promote the use of antidepressant medications. But this dichotomy between depression as a response to environmental stresses versus brain pathology is a false one. The active inference framework allows us to understand that environmental factors have important causal influences on brain and bodily processes, which can manifest in the symptoms of depression. We can understand depression as arising because our beliefs about our future social environments have been shaken, and with this change in model confidence, our management of interoceptive processes is set awry. We can also understand that depression can be precipitated by asocial causes, such as chronic pain and inflammation (or for no obvious reason at all), and that these then influence behaviours and engender changes in the social environment.

Our treatments for depression act at different levels to readjust our generative models. All treatments work at the synapse: either directly, as for antidepressant medications, or indirectly, as for psychotherapies, which act to increase confidence in the psychosocial environment. We should not see these treatments as acting in opposition, but rather in complement. We can only agree that creating an environment that is less depressogenic—where there is stable housing, ability to purchase food and other essential items, and supportive social networks—provides a means of reducing the prevalence of depression. But depression will still occur, as we now see in wealthier people who do not have pressing financial concerns but nonetheless struggle with depression. Depression is a complex disorder, and different treatments will be more or less effective for an individual in ways that are hard to predict. We should consider all effective treatments as operating within the same active inference framework, and understand that there are multiple effective options for alleviating depression. And while we pursue the development of more effective and better tolerated treatments, we should continue to advocate for better communities that do not generate such high levels of depression.

## Data Availability

Data is contained within the article.
